# On chip microfluidic separation of cyclotides

**DOI:** 10.55730/1300-0527.3534

**Published:** 2022-12-20

**Authors:** Reza DIDARIAN, Aliakbar EBRAHIMI, Hamed GHORBANPOOR, Hesam BAGHEROGHLI, Fatma DOGAN GÜZEL, Mohsen FARHADPOUR, Nasrin LOTFIBAKHSHAYESH, Hossein HASHEMPOUR, Hüseyin AVCI

**Affiliations:** 1 Department of Biomedical Engineering, Ankara Yıldırım Beyazıt University, Ankara, Turkey; 2 Cellular Therapy and Stem Cell Production Application and Research Center (ESTEM), Eskişehir Osmangazi University, Eskişehir, Turkey; 3 Department of Chemistry, Faculty of Basic Sciences, Azarbaijan Shahid Madani University, Tabriz, Iran; 4 Department of Plant Bioproducts, National Institute of Genetic Engineering and Biotechnology (NIGEB), Shahrak-e Pajoohesh, Tehran, Iran; 5 Department of Tissue Engineering, School of Advanced Technologies in Medicine, Tehran University of Medical Sciences, Tehran, Iran; 6 Department of Metallurgical and Materials Engineering, Eskişehir Osmangazi University Eskişehir, Turkey; 7 Department of Biomedical Engineering, Eskişehir Osmangazi University, Eskişehir, Turkey; 8 Translational Medicine Research and Clinical Center (TATUM), Eskişehir Osmangazi University, Eskişehir, Turkey

**Keywords:** Microfluidic separation, cyclotide, *Viola ignobilis*, Vigno 5, liquid-liquid extraction

## Abstract

Cyclotides as a cyclic peptide produced by different groups of plants have been a very attractive field of research due to their exceptional properties in biological activities and drug design applications. The importance of cyclotides as new biological activities from nature caused to attract researchers to develop new separation systems. Recent growth and development on chip-based technology for separation and bioassay especially for anticancer having sparklingly advantages comparison with common traditional methods. In this study, the microfluidic separation of Vigno 1–5 cyclotides extracted from Viola ignobilis by using polar and nonpolar forces as a liquid-liquid interaction was investigated through modified microfluidic chips and then the results were compared with a traditional counterpart technique of high-performance liquid chromatography (HPLC). The traditional process of separating cyclotides from plants is a costly and time-consuming procedure. The scientific novelty of this study is to accelerate the separation of cyclotides using modified microfluidic chips with low cost and high efficiency. The results revealed that a novel and simple microfluidic chip concept is an effective approach for separating the Vigno groups in the violet extract. We believe that the concept could potentially be utilized for further drug development process especially for anticancer studies by coupling bioassay chips as online procedures via reducing in time and cost compared with traditional offline methods.

## 1. Introduction

Cyclotides are small bioactive peptides isolated from plants with a head-to-tail cyclic backbone and three conserved disulfide bonds that form a cyclic cystine knot motif and 28–37 amino acids in size [1]. This unique three-dimensional structure gives them inherent stability to resist chemical, enzymatic, and thermal degradation [2]. Due to their unique topological structures, cyclotides have been reported to have a broad range of bioactivities including antimicrobial, anticancer, antivirus, uterotonic and immunomodulatory effects, etc. Thus, these properties, together with recent advances in cyclotides applications as drug design framework, have made them attractive in chemical biology, medicine, drug development and protein engineering [3–8].

Cyclotides are found in plants of the families *Violaceae*, *Rubiaceae*, *Cucurbitaceae*, *Fabaceae*, and *Solanaceae*. Most cyclotides discovered to date have been isolated from plant species *Rubiaceae* [9, 10] or *Violaceae*.[11] Cyclotides in *Violaceae* are almost everywhere and have been found in more than 35 species of this family that have been screened [12–15]. The plant species *Viola ignobilis,* from *Violaceae* family, has been reported to have ten Vigno cyclotides named Vigno 1–10. The Vigno 1–5 and varv A (six peptides) are from Möbius cyclotides subfamily and have high amounts in plant extract which eluted in reverse phase chromatography within 4 peaks with a big challenge in their separation [16]. In this study, Möbius subfamily cyclotides from *Viola ignobilis* were selected as a model for separation by microfluidic chip.

One of the long-term goal of medical researchers is to study natural products with polymers and discover new compounds with biological activities for potential solutions to treat diseases [17–22]. The discovery of cyclic peptides is one of the most promising areas, and researchers, for example, are currently developing the clinical case of a cyclotide volunteer for multiple sclerosis [23, 24]. Cyclotides have anticancer properties and can be engineered to bind and inhibit specific cancer targets. In addition, some cyclotides are toxic to cancer cells although little is known about their mechanism of action [25].

Current techniques for cyclotide screening in plants include extraction, purification, HPLC hydrophobic leaching, and mass range. After identifying the plants carrying cyclotides, the separation and characterization are performed by hydrophobic chromatography and mass spectrometry, respectively. Cyclotide extraction is one of the key steps in the screening step. If the extraction method is performed with high efficiency and purity, it will lead to more successful screening. To address the challenges, the present study sought to develop a simple, rapid, and an inexpensive technique for the extraction and separation of cyclotides from the plant extracts. One of the hot topics of separation science is lab-on-chip microfluidic technology as miniaturization platform which affording faster and sensitive separation, high throughput analysis and significant reducing of samples, solvents and reagents consumption. Microfluidic systems are highly ordered and nonturbulent; fluid flow chips are usually used in controlled biological assays. Microfluidic systems are designed for the utilization of fluid flows with the scales of micrometer range. Fluids in microfluidic systems with microliters flows behave completely different compared with normal milliliters scales [26]. In comparison with conventional separation and chromatographic methods, microfluidic chips have some advantages that make them more suitable. High-resolution separations and detections capability are obtained by using the small component size. Reducing detection limitations makes it possible to achieve extreme sensitivity [27]. The array and multichannel design paves the way for achieving rapid high throughput analysis by decreasing time and cost meaningfully [28, 29]. Microfluidic-based liquid chromatography is used to perform on-chip separation of various types of analytes and their analysis (and in genomics, proteomics, biomarker discovery) [30–33]. Features of microfluidic chromatography are good reproducibility and low sample consumption. The ease of use and low production cost make microfluidic devices attractive for cyclotides screening. On the other hand the microfluidic separation chips have capability for integration with chip based three-dimensional cell culture model which mimic heterogeneous tumors to test toxicity and anticancer activities of drugs [34–36]. Therefore, by the considering of the importance of cyclotides, application of microfluidic chips in separation of cyclotides is inevitable.

**Figure 1:**
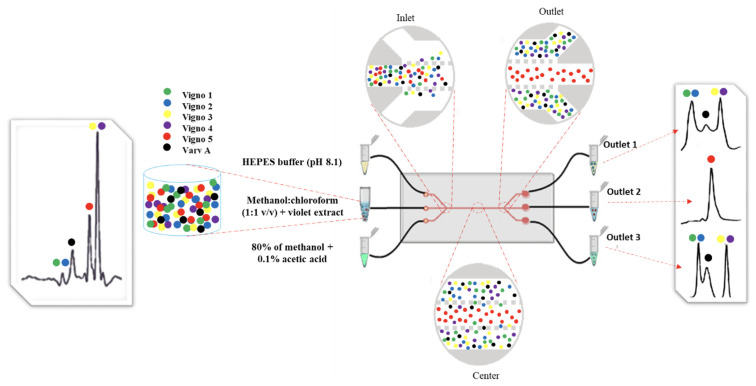
Schematic view of the cyclotides on chip separation. Introducing of HEPES buffer (pH about 8.1) for the first inlet; methanol and (1:1 v/v) chloroform solutions with violet extract for the second inlet; 80% of methanol solution with 0.1% acetic acid for the third inlet.

An approach based on the designing of a microfluidic chip platform developed by PDMS that enables effective separation of Möbius cyclotides from *Viola ignobilis* by using polar and nonpolar forces as a liquid-liquid interaction at low cost, high speed and high efficiency is presented in this study. Cyclotides were continuously separated into three groups on the chip while treated with weak acidic and basic solutions throughout the microfluidic channel (Figure 1). The collected samples from microchannels were analyzed by HPLC to confirm the separation of cyclotides. The obtained results propose chip-based separation which can be used for drug discovery and bioassay as a high throughput screening of cyclotide-carrying plants by off-line coupling to mass spectrometry techniques [37]. We presume that the chances of discovering cyclotides with interesting biological activity and using them against various diseases will increase in the field.

## 2. Materials and methods

### 2.1. Plant collection

In this study, *Viola ignobilis* a member of *Viola* genus from *Violaceae* plant family was collected in the spring 2019 from Negarestan village in the north part of East Azarbaijan province of Iran at an altitude of about 1750 m. After identification of plant species by Dr. Mostafa Ebadi (a plant systematic researcher) at the biology group in Azarbaijan Shahid Madani University, different aerial parts of plant were isolated and dried in shade and stored for the next analyses. On the other hand, thirteen cyclotides were reported in the literature for cyclotides which ten of them were new and reported by Hashempour et al. [7]. In addition, this plant has six cyclotides which eluted in 50% ethanol from solid phase extraction, purification step (SPE) as a fraction.

### 2.2. Extraction and preparation of cyclotides

The cyclotides containing extract was obtained by low voltage electric field extraction method [38]. Briefly, one gram of aerial parts of plant was powdered and extracted by 45 mL acetonitrile/water/formic acid (50:50:0.01%) in extraction chamber with 20 Volte’s electric field. The liquid extract after filtering and isolating from plant materials was evaporated by rotary evaporator and concentrated by freeze-dryer for obtaining of the crud extract. The crud extract was dissolved in ammonium bicarbonate buffer (pH = 8.1) and loaded on solid phase extraction (SPE) C18 cartridge for purification. The loaded extract was eluted by 20 and 50% aqueous ethanol. The eluted fraction by 50% ethanol (%99.99, Merck) was selected as Möbius contains which concentrated by freeze-dryer to obtain crud cyclotides (named violet fraction) and stored at −4 °C for further analysis.

### 2.3. HPLC and MALDI-TOF analysis

The first purpose of HPLC analysis was for confirming and detecting of cyclotides in violet fraction (50% ethanol elution from SPE). The second aim was the monitoring of chips outlets samples by comparing with the peaks pattern of violet fraction in the HPLC chromatogram. A gradient mobile phase system was applied by a RIGOL (China) L-3245 quaternary pump with a L3500 UV detector on a reversed phase C18 column (4.6 × 250 mm, 5 μm, 100 A°). The mobile phase consisted of solvent A (water/0.05% TFA) and B (acetonitrile/0.05% TFA). Gradient elution system was applied as follows; 5% B (0–5 min), 5%–20% B (5–15 min), 20%–55% B (15–20 min), 55%–100% B (30–35 min), 100% B (35–40 min), 5% B (40–40.1 min) and 5% (40.1–45 min). The UV detector wave length was set on 218 nm. An ABSciex 4800 matrix-assisted laser desorption ionization (MALDI-TOF/TOF) system (USA) was used for mass analysis of violet fraction with the mass range of 2000–4000 Da by monoisotopic mass accuracy for the detection of cyclotides.

### 2.4. Production of microfluidic chips

Microfluidic chips were made of polydimethylsiloxane (PDMS) using classical optical and, soft lithography method [39, 40]. 5 inch Si wafer placed on spin coater (Laurell, Model WS-650MZ-23NPPB, USA) and SU8 photoresist (Micro Chem, USA) add on the surface of the wafer and spin coated at 500 rpm for 10 s and then 3000 rpm for 25 s to coating a layer of photoresist on silicon wafer and then exposure to UV light under a photomask to prepare desired microchannel design on photoresist. After the baking process template was produced, and then PDMS mold was fabricated using soft lithography method [41, 42]. A mixture of PDMS and crosslinker (Sylgard 184 silicon elastomer base and Sylgard 184 silicon elastomer curing agent, Dow Corning, USA) was mixed in a ratio of 9:1 (in this study we used the low molecular weight Sylgard 184 PDMS polymer (Component A: Mn = 5.8 × 10^3^ Da; Component B: Mn = 7.5 × 10^3^ Da) [43–45]. AAfter stirring the mixture for 3 min, it was poured onto template and then vacuumed in a desiccator for 20 min to remove bubbles. To harden the PDMS microchip, the template was left at 50 °C overnight, and then the microfluidic chip was formed by peeling off the PDMS layer from the template surface. The PDMS mold was then bond to a glass slide using a custom made plasma device upon oxygen plasma exposure [43]. In this study, a modified method in which the basic phase was injected from the first inlet of the 3-channel chip, the organic phase from the second (middle) inlet, and the acidic phase from the third inlet [46]. The goal here is to dissolve the cyclotide mixture in the organic phase and to interact with the pores between the channels and ensure that the cyclotides pass through the phases. To prevent the effect of acidic and basic solution on the structure of cyclotides, weak acidic and basic solutions were used.

## 3. Results and discussion

### 3.1. Identification of cyclotides in plant extract

**Figure 2:**
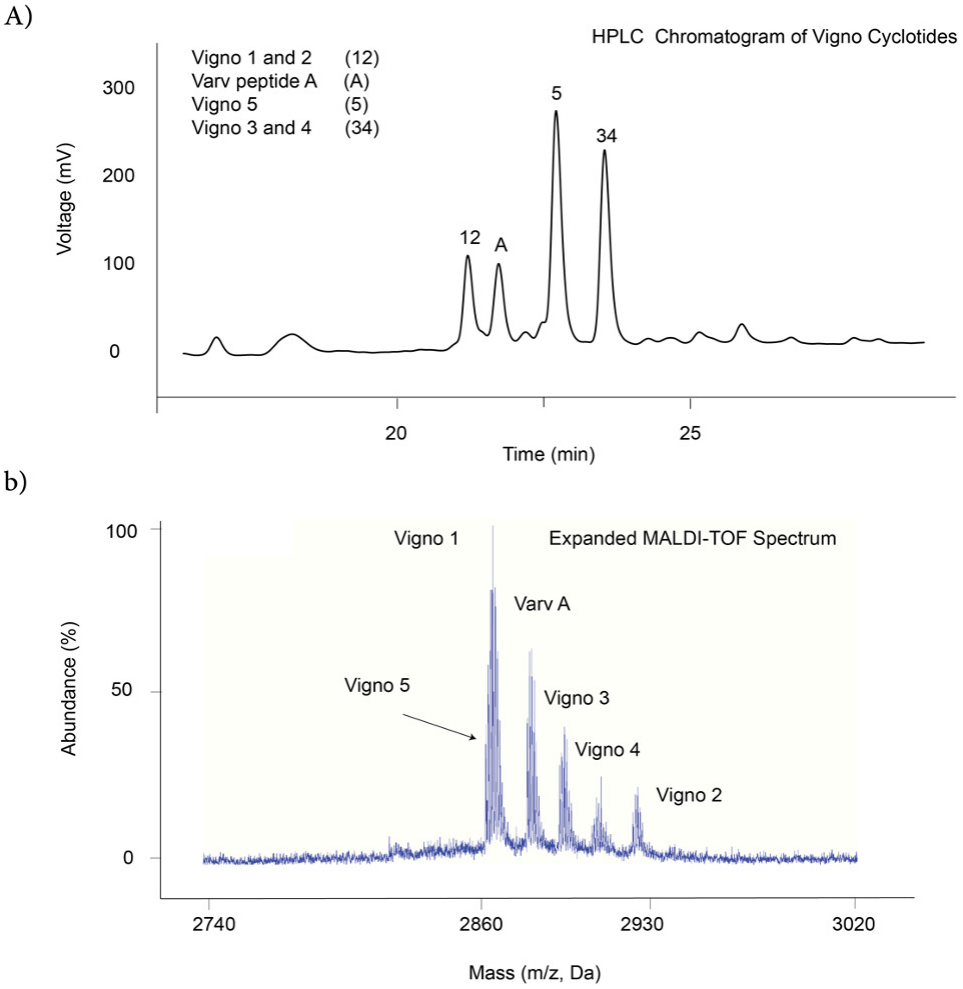
**a)** HPLC and **b)** MALDI-TOF analysis for the violet extract.

The existence of cyclotides in plant extracts is confirmed by two steps including their peaks elution in reverse phase HPLC (25%–55% acetonitrile) and their mass range (2500–4000 Da in MALDI-TOF) [47]. Before the PDMS-made microfluidic chip platform for separation, the crude extract of Möbius cyclotides was purified by SPE C18 cartridge and eluted in 50% ethanol. The eluted fraction was analyzed by HPLC and cyclotides resolved peaks detected in retention time of 20–25 min which related to 32%–44% of acetonitrile in mobile phase (Figure 2a). For identification of cyclotides mass range, MALDI-TOF analysis was applied and their mass weights detected between 2800 and 3000 Da (Figure 2b) in accordance with Möbius cyclotides of *Viola ignobilis* (Table 1) [16].

### 3.2. Designing of microfluidic chips and Möbius cyclotides separation

**Figure 3:**
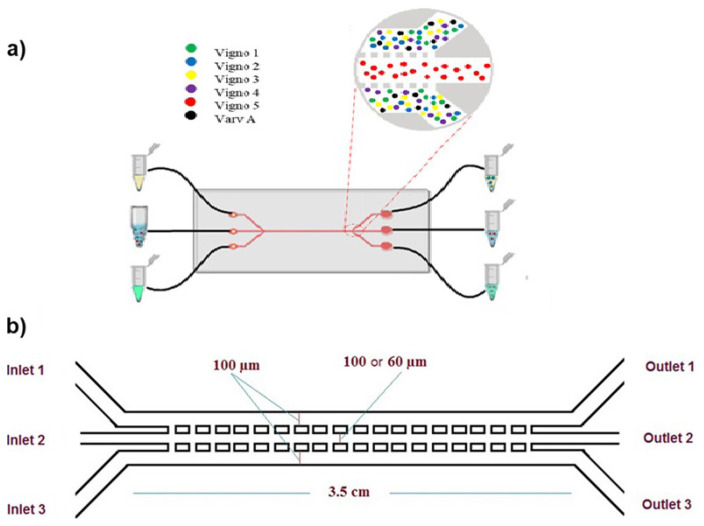
**a)** Schematic view of the chip for target separation. **b)** Microfluidic chips designed for separating cyclotides with different channel widths.

In this study, we aimed to investigate separation of cyclotides based on polarity from violet fraction through a microfluidic PDMS chip while treated with weak acidic and basic solutions throughout the microfluidic channels (Figures 3a and 3b). The three-phase microfluidic chip principle is showed in Figures 3a and 3b, where polar Cyclotides (some polar and nonpolar compounds are present as impurities) are extracted from an organic phase to acidic and basic phases and nonpolar Cycotide (Vigno 5) is remained organic phase. The three-phase microfluidic chip principle is reported by Tetala et al. for separation of polar and nonpolar alkaloids [46]. The PDMS chip contained three interconnected microchannels with three inputs and three outputs. We were inspired by the research of Tetala et al. for the chip design [46]. Along the chip, two series of pillars in size of 120 μm * 70 μm spaced between the lateral channels and middle channel. In each series distance between the pillars are 120 μm. Microchip was designed in two generation with a length of 3.5 cm. Firstly, we applied the dimensions of the chip which reported by Telata et al. [46]. In the first generation, the dimensions of the microchannel were 100 μm (width) and 40 μm (depth). Then, we observed that acidic and basic phase in the side channels diffuse to middle channel (organic phase). To solve this problem, we applied two-fold flow rate in the middle channel in comparison to the side channels. In addition, we decreased the width of the middle channel for the second generation. In the second generation, the dimensions of each microchannel were approximately 100 μm (width) and 40 μm (depth) for the two lateral channels, and it was 60 μm (width) and 40 μm (depth) for the middle channel (Figure 3b). Regarding to the abovementioned optimization procedures there was no diffusion observed from the side channels to the middle channel. The optimized design was obtained when we used the second-generation chip with a middle channel diameter of 60 μm and the flow rate of 3 μL/min for inlets 1 and 3, and 6 μL/min for the second inlet.

During the experiments, HEPES buffer (BioPerformance Certified, ≥99.5% (titration), Sigma-Aldrich, Germany) (pH about 8.1) for the first inlet, 50% of methanol (puriss. p.a., ACS reagent, reag. ISO, reag. Ph. Eur., ≥99.8% (GC), Sigma-Aldrich, Germany) and chloroform (Chloroform RS, Carlo Erba, France) solutions, and violet fraction for the second inlet; 80% of methanol solution with 0.1% acetic acid (puriss., meets analytical specification of Ph. Eur., BP, USP, FCC, 99.8%–100.5%, Sigma-Aldrich, Germany) for the third inlet were injected. Then, the separation was performed with different flow rates (Table 2). Due to the high viscosity of the solution injected from the middle inlet (methanol + chloroform + violet fraction), the speed of the solution inside the channel was slow. Therefore, the flow rate of the intermediate inlet solution was set to twice the flow rate of the lateral inlets solutions. Finally, the samples were collected from the three outlets and then analyzed using by HPLC.

**Figure 4:**
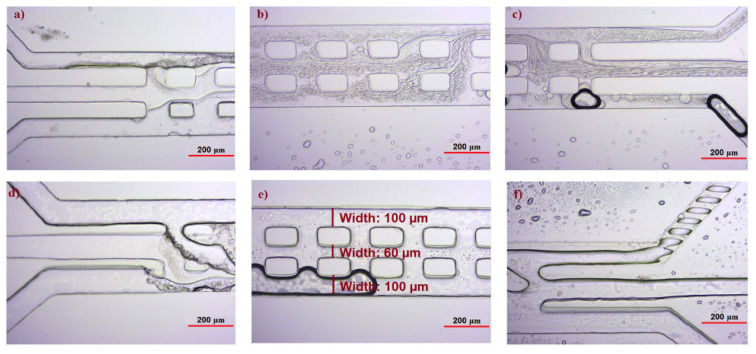
Photographs taken during separation studies; **a), b)** and **c)** show the chip design with the same channel widths (100 μm); **d), e)** and **f)** show the chip design with different channel widths (100 μm for the two lateral channels, and 60 μm for the middle channel). As it can be seen, the liquids can interact through the pores.

During the analysis, the flow behavior of liquids and separation by microscope were investigated and photos were taken to observe the fluids interaction with each other (Figure 4).

As previously indicated, the microfluidic chip was designed in two models. In the first model, the widths of all three microchannels were kept the same. Due to the difference in viscosity between the solutions, a drop in pressure in the inlet 2 (middle channel) was observed. In this case, probably the interaction was not made; thus, the transfer of cyclotides did not occur. To compensate for the pressure drop, the second model chip was designed in which the pressure drop was partially compensated by reducing the diameter of the middle channel. Following the experiments and testing different flow velocities, optimum results were obtained when the second model chip and the flow rate of 3 μL/min were used for the first and third inlets, and 6 μL/min was used for the second inlet.

**Figure 5:**
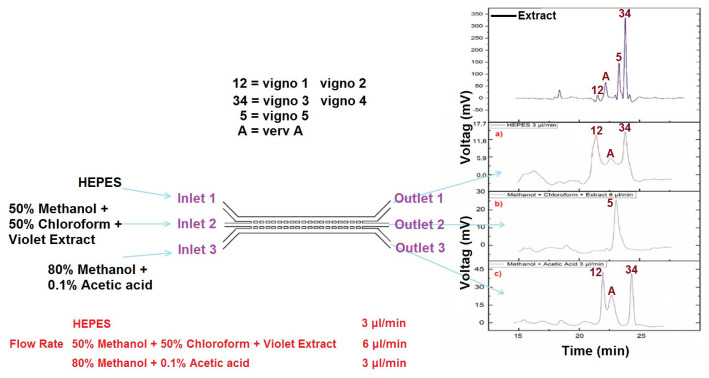
HPLC analysis results for violet extract and the sample collected from 3 outlet: **a)** violet extract, **b)** sample collected from outlet 1, **c)** sample collected from outlet 2, and **d)** sample collected from outlet 3.

All samples collected from each microfluidic chip outlets were also analyzed by HPLC during various experiments. Figure 5 shows the HPLC results of samples collected from three microfluidic chip outlets. In this case, Figure 5a depicts the results of HPLC analysis for violet extract; there are four peaks for the violet fraction (Vigno 1, 2, 3, 4, 5, and varv A), indicating the presence of five cyclotides. Figures 5b, 5c and 5d showed that different peaks were seen for three microchip outlets. Despite injecting a solution containing violet extract from the second inlet, there was only one peak (Vigno 5) in the second outlet (Figure 5c), and the other cyclotides were transferred to the other two outlets. Both Vigno 1 and Vigno 2 in peak 12 and both Vigno 3 and Vigno 4 in peaks 34 are coeluted. Therefore, they could not be separated from each other.

Figure 5b shows the HPLC results for the sample from outlet 1. Figure 5b displays two different peaks (Vigno 12 and 34) at the outlet of the first channel. Figure 5d shows the HPLC result for outlet 3. There were two peaks (Vigno 34, A and 12) at outlets 1 and 3, showing the cyclotides transferred from channel 2 to channels 1 and 3.

The repeated experiments on separation of cyclotides in violet fraction showed similar results, as shown in Figure S1. Therefore, it can be concluded that the separation was reproducible using proposed microfluidic chips. The peaks of Vigno 1 and Vigno 2 are very close to each other, just like Vigno 3 and Vigno 4; therefore, exact separation of these groups could not be performed.

Separation of cyclotides mixture by microfluidic systems is easy to use, have low production costs, and do not require trained personnel. The scientific novelty of this study was application of microfluidic in the separation of cyclotides for the first time. Due to the small size of the chip and the use of microliters of samples and reagents, the cost is reduced, resulting in significant savings in the total separation. Microfluidic systems are highly ordered and nonturbulent and therefore have high efficiency. This kind of microfluidic separation system can be integrated by bioassay chips or organ-on-chip models for screening of cyclotides in drug design and discovery [48–51]. In addition, the microfluidic chips can be coupled to mass spectrometry devices in off-line mode by collecting of chips outlets which can be used for identification purposes as hyphenated system. This application can be useful for new cyclotides discovering from plant sources by considering of high throughput screening capability and low cost and time consumption [47, 52].

## 4. Conclusion

Cyclotides mixtures from *Viola ignobilis* were successfully separated using a simple modified three-channel microfluidic chip based on polar and nonpolar interaction. For this purpose, two different chips were designed and then were produced by photolithography. First, a 3-channel chip with the same width was designed for polar and nonpolar separation. In this case, difference in polarity of cyclotides by sending different solvents of basic, organic, and acidic solutions were used. Due to the high viscosity of the intermediate solution, the interaction of the solutions increases as the width of the intermediate channel decreases; therefore, the second version chip in which a 3-channel with different widths was designed. As shown in the results, the separation was carried out at different flow rates. When a low flow rate is used, their interaction is reduced due to the difference in viscosity of the solutions and as a result the separation is not well done. On the other hand, if the flow rate is high, the interaction of the liquids is reduced and the molecules do not have time to pass between the phases and therefore separation cannot be made. The best results were obtained when we used the second design with a flow rate of 3 μL/min for inlets of 1, 3; and 6 μL/min for inlet 2. At these stages, the violet extract as cyclotides mixture was dissolved in the organic phase. Due to the presence of pores between the channels, cyclotides were transferred through the phases while the solutions interacted. The samples were collected from the outlets and then analyzed by HPLC. HPLC results showed that Vigno 5 was in the sample collected from outlet 2 and Vigno 1, 2, 3 and 4 in the sample collected from other outlets. The results clearly showed the transfer of cyclotides from the organic phase to other phases. As shown in Figure 4, some bubbles have formed inside the channel. Considering the repetition of experiments and obtaining similar results, and since Vigno 5 has been completely separated in all experiments it can be concluded that the presence of bubbles does not affect the separation performance. Based on the obtained results, it was proved that the separation process of cyclotides with this method can be performed at a certain flow rate and differences in polarity. HPLC method [7, 16] is a conventional method and extraction with the help of a microwave [53] is a relatively new method for separating cyclotides from the plant extracts, but only a mixture of cyclotides can be obtained by using these methods. Then, it is necessary to use the MALDI-TOF method to separate the individual cyclotides from this mixture. These methods are expensive, time-consuming, and require skilled personnel to implement. Based on the results, it can be concluded that our designed system can be a promising platform for the rapid and precise separation of cyclotides from the extract without the need for precision instruments, sophisticated working methods, and skilled personnel for the separation.

Figure S1 HPLC analysis results for violet extract and the sample collected from 3 outlet: a) sample collected from outlet 1, b) sample collected from outlet 2, and c) sample collected from outlet 3.

## Figures and Tables

**Table 1:** Möbius cyclotides from *Viola ignobilis* sequences and mass weights.

Name	Sequence alignment	Mass weight (Da)
Vigno 1	G-LPLCGETCAGGTCNTP--GCSCS-WPVCVRN	2860.1
Vigno 2	GSSPLCGETCAGGTCNTP--GCSCS-WPVCVRD	2922.1
Vigno 3	G-LPLCGETCAGGTCNTP--GCSCS-WPVCTRN	2890.1
Vigno 4	G-LPLCGETCAGGTCNTP--ACSCS-WPVCTRN	2904.1
Vigno 5	G-LPLCGETCAGGTCNTP--GCSCG-WPVCVRN	2858.1
Varv A	G-LPVCGETCAGGTCNTP--GCSCS-WPVCTRN	2876.1

**Table 2:** Use of different flow rates (μL/min) for on-chip experiments.

Inlet 1	25	10	4	3	2.5	1.5	8	5	3	2.5
Inlet 2	50	20	8	6	5	3	8	5	3	2.5
Inlet 3	25	10	4	3	2.5	1.5	8	5	3	2.5
