# Supplementary figures and images for: On chip microfluidic separation of cyclotides
Source: Turk J Chem. 2022 Dec 20;47(1):253–62. doi: 10.55730/1300-0527.3534 (PMC10504020; doi:10.55730/1300-0527.3534)

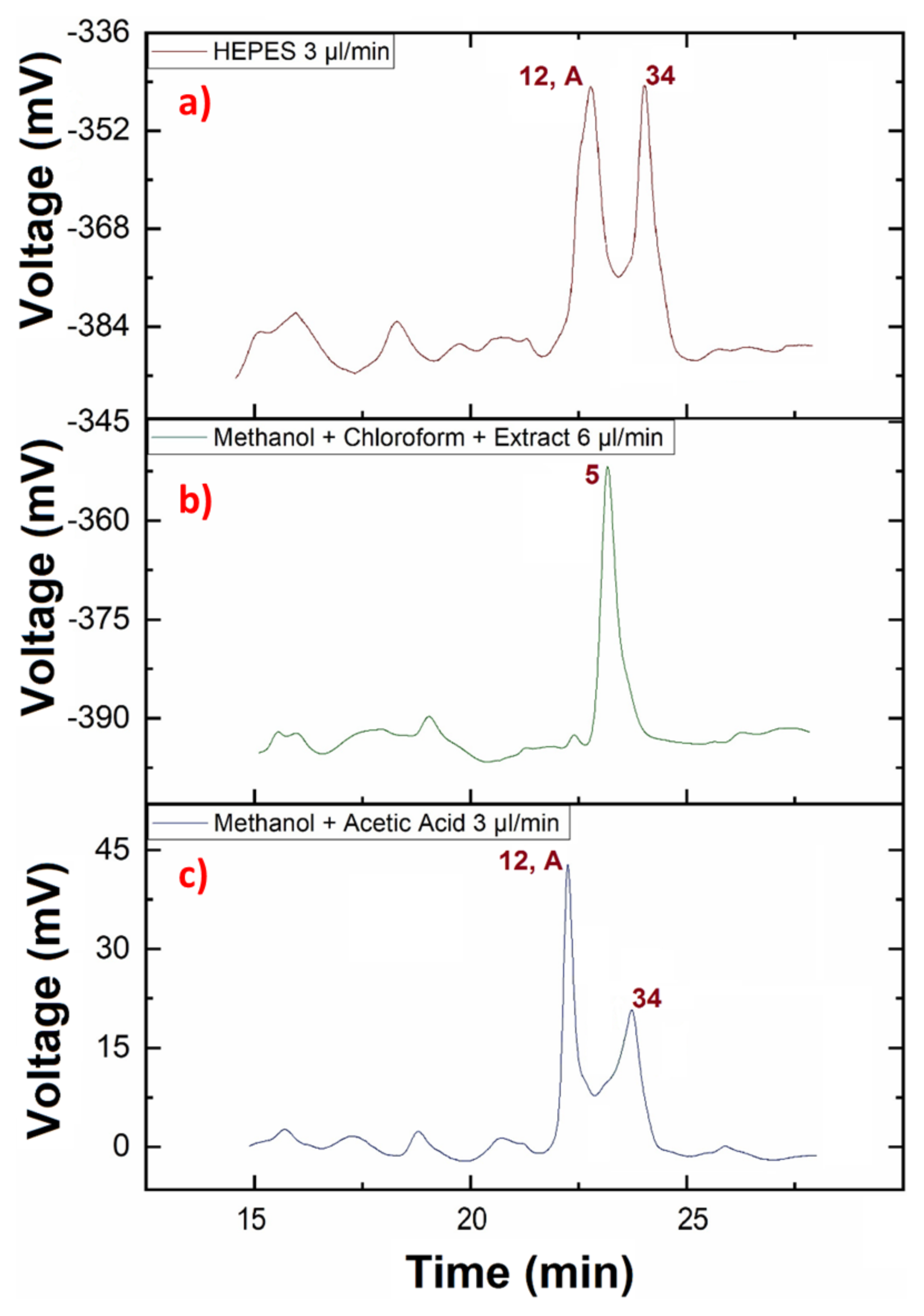

Supplement: Figure S1 — HPLC analysis results for violet extract and the sample collected from 3 outlet: a) sample collected from outlet 1, b) sample collected from outlet 2, and c) sample collected from outlet 3. [file turkjchem-47-1-253s1.tif]
